# The Role of Intelligence in Social Learning

**DOI:** 10.1038/s41598-018-25289-9

**Published:** 2018-05-02

**Authors:** Alexander Vostroknutov, Luca Polonio, Giorgio Coricelli

**Affiliations:** 1 Center for Mind/Brain Sciences, University of Trento, Trento, Italy; 2 Department of Economics, University of Southern California, California, USA

## Abstract

Studies in cultural evolution have uncovered many types of social learning strategies that are adaptive in certain environments. The efficiency of these strategies also depends on the individual characteristics of both the observer and the demonstrator. We investigate the relationship between intelligence and the ways social and individual information is utilised to make decisions in an uncertain environment. We measure fluid intelligence and study experimentally how individuals learn from observing the choices of a demonstrator in a 2-armed bandit problem with changing probabilities of a reward. Participants observe a demonstrator with high or low fluid intelligence. In some treatments they are aware of the intelligence score of the demonstrator and in others they are not. Low fluid intelligence individuals imitate the demonstrator more when her fluid intelligence is known than when it is not. Conversely, individuals with high fluid intelligence adjust their use of social information, as the observed behaviour changes, independently of the knowledge of the intelligence of the demonstrator. We provide evidence that intelligence determines how social and individual information is integrated in order to make choices in a changing uncertain environment.

## Introduction

Learning is an important and flexible process that allows humans to adapt to their environment. A first basic source of learning is personal experience. Humans interact directly with the environment and learn from the feedback they receive. A second source of learning is observing other people interacting with the same environment. In a world where we need to adapt quickly to ever-changing circumstances (e.g., climate fluctuations, socio-political commotion), the ability to learn from others is fundamental because it allows us to foresee the consequences of our actions without experiencing them directly. However, people should be selective in which situations they rely on social learning strategy as it can be efficient in some cases and inefficient in others^1–7^. An efficient social learning strategy should specify under which circumstances to pay attention to social information, which individual to imitate, and the type of information that should be taken into account^8,9^. The most studied classes of social learning strategies include: *frequency*-*dependent rules*, such as conformity or anti-conformity to the most chosen alternative^10–13^; *payoff*-*based rules*, where the level of imitation depends on the payoffs achieved by a demonstrator in the recent past^14,15^; *confidence*-*based rules*, when confidence of individuals and demonstrators modulates imitation^13^; and *prestige*-*based rules*, where the level of imitation depends on the status of the observed other^16–19^.

Another aspect is how the integration of social and individual information is accomplished^20,21^. In an environment where demonstrators are observed repeatedly, on the one hand, it is possible to learn from others using simple reinforcement learning^22^, which is the case when an agent imitates others, evaluates the feedback she receives after imitation, and chooses whether to keep imitating or not, depending on the outcome^23–27^. On the other hand, a more strategic use of social information involves understanding the rationale behind the observed choices^6,13,28–30^. This is a more sophisticated mechanism of learning that requires the agent to integrate what she has observed with the feedback she has directly received from the environment. The adoption of this integrated learning process can be more efficient, especially when the environment is changing or when the expertise of the demonstrator is unknown, but is also costlier since it requires a higher level of attention and an ability to understand and integrate signals coming from different sources. A key question is, thus, when and how to switch between social and individual learning. Experimental evidence shows that individuals increase their level of imitation with task difficulty and cost of individual learning, and decrease it with the probability of changes in the environment^13,31^. The tendency to use social learning is also related to the cognitive abilities of the individual. In particular, individuals with low intelligence scores use social (instead of individual) learning more often, as compared to the individuals with high intelligence scores, who, in addition, have a higher ability to understand when and whom to copy^32^.

The goal of this study is to determine how variation in fluid intelligence of participants and demonstrators modulates the use of social information and employment of different social learning strategies. In particular, we are interested in how individual characteristics related to fluid intelligence influence imitation decisions. To achieve this, we study behaviour of participants in a complex, stochastic, and unstable environment while they are observing another individual choosing in the same setting^33,34^. The task was designed so that individual learning requires effort and, more importantly, it is hard to recognise the competence of the observed model from her choices alone. To see how reliance on social information and rate of copying the demonstrator change, we have participants observe the actions of either a highly competent or less competent other, and vary the availability of the information about her fluid intelligence. In addition, the dynamic nature of the task and multiple imitation choices that each participant has to make allow us to investigate in detail how social and individual information is integrated.

Evidence from experimental economics literature on strategic thinking suggests that the cost of learning in interactive settings varies with fluid intelligence^35–40^. Studies in cultural evolution literature find a variation in social learning strategies that depends on social group and individual characteristics^13,32,41,42^. Having these findings in mind, we hypothesize that low fluid intelligence participants have a relatively high cost of learning, which implies that it should be difficult for them to perform efficiently in the task and, as a consequence, hard to interpret the observed actions of the demonstrator when her competence is unknown. This should lead to low confidence, low efficiency, and, as a result, strong dependence of the imitation rate on the information about the demonstrator’s intelligence (prestige bias). Behaviourally, we should, thus, observe (1) relatively low earnings; (2) inability to modulate imitation rate with changing characteristics of choices of the demonstrator; (3) no difference in imitation rates between competent and less competent demonstrators when their intelligence is unknown; (4) stable increase in imitation when the intelligence of the competent demonstrator is known (and, as a result, increase in earnings). High fluid intelligence participants, who have low cost of learning, should be able to learn well in the task and also able to recognise the competence level of the demonstrator from her actions even if her intelligence is unknown. This should lead to high confidence, high efficiency, and dependence of the imitation rate on the dynamic features of the demonstrator’s choices instead of the information about her intelligence (no prestige bias). For high fluid intelligence participants we should, thus, observe (1) relatively high earnings; (2) dependence of the imitation rate on the changing properties of the observed choices; (3) different imitation rates of competent and less competent demonstrators when their intelligence is unknown; (4) no difference in imitation rates of a competent demonstrator when her intelligence is known vs. when it is not.

**Figure 1:**
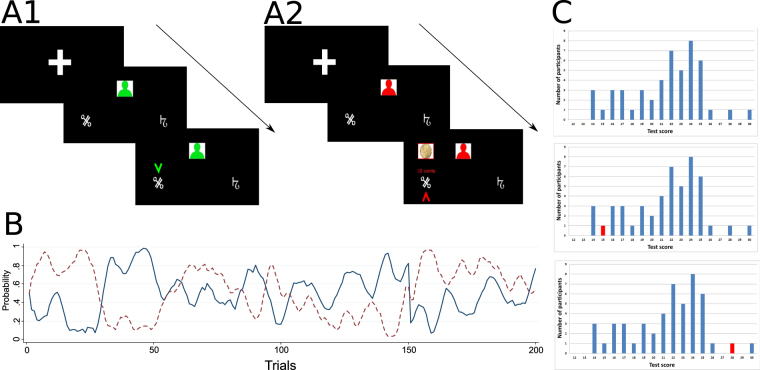
Experimental design. (**A**) Participants choose between two options which can give them a 10 cents reward with some probability (A2). In each trial, a red figurine, presented after a fixation screen, informs participants that it is time to choose. In Experiment 1 participants choose without observing anyone’s choices. In Experiments 2 and 3, periodically (6–12 trials in a row, for a total of 100 out of 200 trials across the experiment), and before their choice, participants can observe the choice of another participant who took part in Experiment 1 (A1). The observed other is represented by a green figurine and her choice is shown with a green tick. Participants do not observe the outcome of that choice. (**B**) Throughout the 200 trials of the experiment the probabilities of reward from the two arms change according to prespecified random processes (the two lines on the graph). It was carefully explained to participants in Experiments 2 and 3 that these probabilities were the same for them and the demonstrator they observe. (**C**) Participants in Experiments 2 and 3 received different information on the observed other. In the NovisHigh and NovisLow treatments they see only the distribution of the Raven scores of potential demonstrators (graph at the top). In the VisHigh and VisLow treatments they see the distribution and the Raven score of the other they observe marked by a red bar (graph in the middle and at the bottom). In Experiment 2 all participants know their own Raven score. In Experiment 3 participants took the Raven test before the main task, but were not informed about their Raven score (everything else was as in Experiment 2).

To test our hypotheses we use a two-armed bandit problem in which the probabilities of reward from the two arms are determined by two independent stochastic processes. In each trial, participants choose one of the two arms, which gives them a fixed reward with some probability (Fig. 1A2). The probabilities of reward from the two arms change over time as shown in Fig. 1B. Participants were not informed about the exact processes guiding the probability changes, but they knew that these probabilities change gradually. In Experiment 1, participants make their decisions in 200 trials without the possibility to learn from others. In Experiments 2 and 3, participants are presented with exactly the same 200 trials as in Experiment 1, but, from time to time before their choice, they are able to observe the action of a demonstrator (Fig. 1A1). They are aware that this person, whom they observe, was making her choices in a previous experimental session (Experiment 1) and that the probabilities of reward that she faced were the same as the probabilities in the current experiment (Fig. 1B). To assess intelligence, we use a 20-minute version of the Raven Advanced Progressive Matrices Test as a measure of fluid intelligence of our participants^43^. The Raven APM test was found to be a measure of the general ability to think in an abstract way, recognize patterns, reason, and discern relationships, all of which should be crucial for efficient learning from observation in a changing stochastic environment. Before starting the task, participants in Experiments 2 and 3 were provided with the histograms of the Raven APM scores (from now, Raven scores) of participants from Experiment 1 (Fig. 1C). Two participants were used to act as demonstrators: a participant with a low Raven score (15 matrices solved correctly) and a participant with a high Raven score (28 correct matrices). Participants were not aware of the relation between the Raven score and the performance of the demonstrator in the learning task.

In order to test the hypothesis that high fluid intelligence participants can recognise the competence of the demonstrator by only observing her actions, whereas low fluid intelligence participants cannot (Hypothesis 1), we run two treatments in which participants observe a low or high Raven demonstrator, but her Raven score is not visible. Participants, included in these two treatments (NovisLow for a low Raven other and NovisHigh for a high Raven other), see the histogram of the Raven scores from Experiment 1 (Fig. 1C top) and are told that the person they observe is one of those represented on it. To test the hypothesis that low fluid intelligence participants react to the information about the Raven score of the demonstrator and high fluid intelligence participants do not (Hypothesis 2), we run two more treatments (VisLow for a low Raven other and VisHigh for a high Raven other) in which the Raven score of the demonstrator is visible to participants. This information is delivered through the same histogram as in the previously described treatments, only now the Raven score of the observed individual is indicated with a red bar (Fig. 1C middle and bottom). To test the hypothesis that high (low) fluid intelligence participants (do not) modulate their imitation with the changing properties of choices of the demonstrator (Hypothesis 3), we introduce in our analysis an observable measure of demonstrator’s confidence—the number of times she switches between actions—and check if the imitation rate is influenced by it dynamically^44^. Finally, to verify that the differences among the four treatments come from fluid intelligence and not from the information about participants’ own Raven score we provide information about one’s own Raven score in Experiment 2, and we do not in Experiment 3 (see Table 3 in Appendix B.1 for the detailed information about all experiments and treatments).

## Results

### Imitation

We start with providing evidence for the hypotheses that are concerned with the reactions of participants to the Raven score of the demonstrator and its visibility (Hypotheses 1 and 2). We analyse the aggregate average levels of imitation in the four treatments of Experiment 2. By *imitation* we mean the situations when a participant chooses the same action as the observed other. There are two types of imitation. The first is *pure imitation*: a participant sees what the observed other has chosen and decides to choose the same action. The second is *coincidental imitation*: a participant chooses the same action as the observed other because she thinks this is the best action to choose regardless of what the demonstrator does (for example when the probabilities of reward from the two arms are very different and it is obvious which arm is better at the moment).

We would like to focus our analysis on the cases of pure imitation. However, in the periods in which participants observe a demonstrator’s choice we cannot tell apart pure imitation from coincidental one. One way to control for coincidental imitation is to notice that, in the periods when the other is not observed, all cases of imitation are coincidental. Therefore, assuming that, on average, coincidental imitation is the same when the demonstrator is observed and not observed, we can use the average rate of imitation in the periods when the demonstrator is not observed as a proxy for the average coincidental imitation when she is observed. Thus, we consider *an adjusted imitation rate* that, for each participant, is equal to the average rate of imitation in periods when the other is observed minus the average rate of imitation when her actions are not observed. This adjustment is necessary to correctly estimate pure imitation since the behaviour of participants is very heterogeneous: the rate of coincidental imitation when the demonstrator is not observed ranges from 0.38 to 0.87.

**Figure 2:**
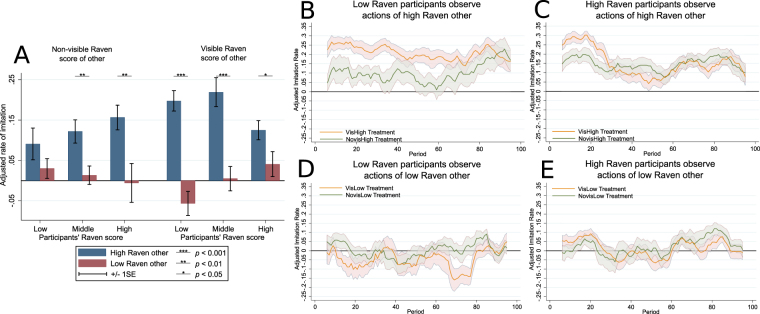
Adjusted imitation rate in Experiment 2. (**A**) The adjusted rate of imitation by the terciles of the Raven score of the participants in treatments with a non-visible and visible Raven score of the other. Blue (red) bars represent the adjusted rate of imitation in treatments in which participants observed the high (low) Raven other. The *p*-values (from left to right: 0.003, 0.004, <0.001, <0.001, 0.033) denote the significance of the *t*-tests on the differences in coefficients of an ordinary least squares (OLS) regression that the bars represent (first column of Table 4 in Appendix B.2). (**B**) The dynamics of the running average of the adjusted imitation rate of low Raven participants (below median Raven score) when they observe a high Raven other (only for 100 periods when the action of the other is observed). Ranges are ±1 SE. (**C**) Same as B only for high Raven participants (above median Raven score). (**D**,**E**) Analogous graphs for the situation when the low Raven other is observed.

Figure 2A shows the adjusted rates of imitation in the NovisHigh, NovisLow, VisHigh, and VisLow treatments averaged by the terciles of the Raven score of participants. We see significant differences in the adjusted rate of imitation between observing high and low Raven demonstrators for all terciles of the Raven score of participants and both a visible and non-visible Raven score of the demonstrator except for Low Raven participants (tercile 1) in the NovisHigh and NovisLow treatments. Middle and high Raven participants (terciles 2 and 3) are able to recognise a competent demonstrator even when her Raven score is unknown, while low Raven participants are not, which provides support for Hypothesis 1. This result shows that fluid intelligence correlates with the ability to understand when copying the demonstrator is worthwhile and corroborates previous findings that high performance demonstrators (in our case high Raven other) are copied more often^13^. We provide an additional angle to these results: when participants are not informed about the competence of the demonstrator (the NovisHigh and NovisLow treatments), they copy the high performance other more often than the low performance one only when they can *recognise* her as such. In our experiment low Raven participants are less able to do that, so their rate of imitation of the high performance demonstrator does not change based on the observed behavior of the demonstrator.

Next, we turn to the analysis of the visibility of the Raven score of the demonstrator (Hypothesis 2). When we compare imitation rates in the VisHigh and NovisHigh treatments we find that low and middle Raven participants increase their imitation when they know that the other is high Raven (increase in imitation: 0.097*, *t*-test *p* = 0.032 and 0.097*, *p* = 0.034 respectively; see Appendix B.2 for details). This supports Hypothesis 2 and suggests that low/middle Raven participants interpret the information about the Raven score of the demonstrator as a signal of competence in the task even though they do not know how much Raven score correlates with it. This is in line with the studies on unconditional copying of successful, knowledgeable, or prestigious models^14,17,45–48^. Conversely, high Raven participants are not significantly affected by the visibility of the high Raven score of the demonstrator (−0.031, *p* = 0.419). Unlike low/middle Raven participants, they do not react to this information but identify a competent demonstrator by her actions. Taken together, we find that intelligence determines the sensitivity to (possibly irrelevant) information about the skills of the demonstrator. Similar differential reliance on learning from models was found in between-cultures studies^42,49^. We add to this literature by showing that variation in social learning strategies can arise from difference in the ability to interpret the actions of the demonstrator, which is correlated with fluid intelligence. It should be also noted that our results are robust when using a different measure of participants’ cognitive ability (see Appendix B.3).

An additional question of interest is whether low Raven participants learn during the experiment and become more like high Raven participants, or whether they maintain their tendency to imitate a high Raven demonstrator more when her Raven score is known? Figure 2B–E show the dynamics of the adjusted imitation rate of low and high Raven participants (below and above the median Raven score). Figure 2B,C illustrate the moving averages in the VisHigh and NovisHigh treatments. Low Raven participants demonstrate a significantly increased rate of imitation when they know that the demonstrator is high Raven, which lasts almost until the end of the experiment as predicted by Hypothesis 2. High Raven participants are affected by this information only in the first 28 periods of observation and then exhibit the same imitation rate as in the NovisHigh treatment. This suggests that high Raven participants learn to understand the meaning behind the choices of the other after about 28 periods of observation, whereas low Raven participants do not and keep relying on the information about the demonstrator’s Raven score. Figure 2D,E show the dynamics of imitation for the low Raven other. In this case, neither high nor low Raven participants change their rate of imitation with the visibility of the Raven score of the demonstrator. The difference in behaviour of high and low Raven participants can be interpreted in terms of difficulty to learn. One possibility is that low Raven participants have a high cost of asocial learning, and it is difficult for them to interpret the observed choices of the demonstrator. Therefore, following the observed other, when her high competence is known, is adaptive for low Raven participants^50^. High Raven participants seem to be able to learn how to perform in the task as it unfolds and become more confident in interpreting the actions of the demonstrator. Thus, they rely on the information about the Raven score of the other only at the beginning of the task. This result supports the evidence provided in previous experiments^13^ that an increase in confidence shifts the balance between social and asocial learning towards the latter.

### Efficiency and Earnings

**Figure 3:**
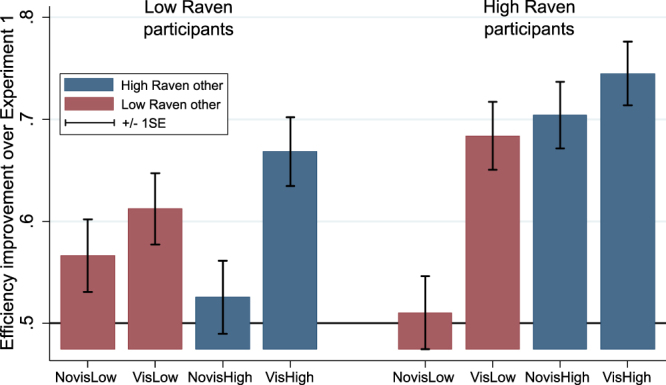
Efficiency improvements over Experiment 1. The bars show the proportion of periods in which the average efficiency in Experiment 2 (by treatment and Raven score of participants) exceeds average efficiency in Experiment 1 (both smoothed by 5-period moving average). The bar colours stand for the Raven score of the observed other. Ranges are ±1 SE.

Next we test the hypothesis that high Raven participants exhibit higher efficiency of choices and earn more than low Raven participants. A choice of a participant is *efficient* if she chooses the action with the highest probability of reward. In our data the lowest efficiency rate is 0.45 and the highest efficiency rate is 0.87, which is a dramatic difference suggesting that some participants are much better at learning the task than others (see Fig. 5 in Appendix B.4 for the distribution). Figure 3 shows the improvement in the efficiency rankings for the four treatments of Experiment 2 as compared with Experiment 1.

The figure shows that the efficiency improvements of high Raven participants are larger than those of low Raven participants in all treatments except NovisLow (improvement of 0.5 is due to chance). To support this finding, we perform non-parametric tests on individual efficiency rates. The Kruskall-Wallis test of the nine groups (participants in Experiment 1 and low and high Raven participants in the four treatments of Experiment 2) shows a significant difference among them (*p* = 0.042). For low Raven participants we can reject the null hypothesis of equal distributions of efficiency rates only for the VisHigh treatment (rank-sum test: *p* = 0.031). For high Raven participants we can reject the equal distributions hypothesis for all but the NovisLow treatment (rank-sum tests: *p* = 0.063 in the VisLow treatment; *p* = 0.018 in NovisHigh; *p* = 0.004 in VisHigh). In support of Hypothesis 3, low Raven participants significantly increase their performance only when they know the Raven score of the high Raven demonstrator, while high Raven participants manage to increase their efficiency in all but NovisLow treatment. This constitutes direct evidence that high Raven participants are able to extract useful information about the environment just by looking at the behaviour of the demonstrator, which confirms the importance of balancing social and individual information^42^.

Participants’ earnings are closely related to efficiency, thus, it is not surprising that we observe similar results. Low Raven participants show significantly higher earnings, as compared to the earnings of the participants in Experiment 1, only when they know that they observe a high Raven demonstrator (rank-sum test: *p* = 0.044). High Raven participants increase their earnings in the NovisHigh and VisHigh treatments (rank-sum tests: *p* = 0.056 - NovisHigh; *p* = 0.002 - VisHigh). This shows that high Raven participants earn more money whenever they observe a high Raven other (with visible Raven score or not) and low Raven participants do so only when they know that they are observing a high Raven other.

Finally, we analyse where the difference in efficiency improvements between low and high Raven participants comes from. We relate it to two characteristics of participants: their Raven score and how often they switch between actions. The latter is a measure of how confident participants are about the expected rewards from the two actions. When expected probabilities of rewards are very different, participants are sure about which action is better. This leads to high confidence and low number of switches between actions. When expected rewards from the two actions are very similar, participants are uncertain about which action should be chosen. Thus, their level of confidence is low and they might switch a lot between actions. The number of switches is also a noticeable feature of the behavior of the demonstrator, which can be taken as a proxy for her level of confidence and, thus, can be utilised in the decision to imitate (see the next section). Table 5 in Appendix B.5 reports the regressions that connect one’s own Raven score, number of switches, and efficiency/earnings. The regressions show that high Raven participants switch less than low Raven participants and also earn more money, and that a high number of switches decreases efficiency and earnings. Therefore, we find an observable behavioural property—number of switches—that is correlated with the Raven score, determines how efficient the asocial learning strategy is, and can potentially signal the confidence level of the demonstrator.

### Strategic Use of Social Information

To support the hypothesis that high Raven participants are more strategic than low Raven ones in their reliance on social information (Hypothesis 3), we analyse imitation choices period by period and test if participants are able to infer the connection between the number of switches of the demonstrator and her efficiency. We estimate a panel logit regression reported in Table 1 (see Table 7 in Appendix B.6 for the linear probability model) where the dependent variable is an indicator of whether a participant has chosen the same action as the demonstrator or not.

**Table 1:** Random effects logit regression of imitation choices in Experiment 2.

Participants:	Prob[imitation = 1]	
	Low Raven	High Raven
visible	0.057	0.274
	(0.317)	(0.238)
value of imitation	1.011***	1.209***
	(0.236)	(0.215)
switches of other	−0.546	−0.664*
	(0.351)	(0.304)
visible × value of imitation	0.118	−0.356
	(0.346)	(0.281)
visible × switches of other	0.216	0.002
	(0.51)	(0.413)
constant	0.388	0.281
	(0.222)	(0.178)
*N* observations	6,095	8,463
*N* participants	67	93

The dependent variable imitation is 1 when a participant chooses the same action as the demonstrator (see Appendix A for the description of all variables used in the analyses). The independent variable visible is 1 if the Raven score of the demonstrator is known to participants. The variable value of imitation tracks the average payoff obtained from choosing the same action as the demonstrator in the last 10 times she was observed. The variable switches of other is equal to the number of switches between actions that the demonstrator made in the last 10 times her actions were observed. Standard errors in parentheses. *p < 0.05; **p < 0.01; ***p < 0.001.

We find that the imitation choices of both high and low Raven participants depend on value of imitation, the variable that tracks how successful imitation was in the recent past: the higher its value, the more participants imitate the other. This finding is not surprising since such behaviour is the simplest and the most natural way of modulating imitation choices. The regression suggests, however, that low Raven participants do not seem to be able to make more complex inferences about the observed choices: they do not use the number of switches of the demonstrator as a signal of her efficiency, and earnings (see Appendix B.5). Conversely, high Raven participants do increase their imitation when they observe that the demonstrator does not switch too often. Thus, in accordance with Hypothesis 3, we conclude that high Raven participants are more strategic than low Raven participants in weighing up social against individual information. In particular, they are able to interpret the number of switches of the demonstrator as a signal of her confidence about the choice and to use it to modulate their imitation. This fits well with the previous findings that the use of social information increases with the confidence of the demonstrator^13^.

### Experiment 3

All previous analysis was based on the data from Experiment 2, where participants knew their own Raven score. It is not inconceivable though that the effects on imitation reported above are caused simply by this information and not by the fluid intelligence. In order to show that this is not the case, we ran Experiment 3 that is the same as Experiment 2 in all respects except that participants were not informed about their own Raven score. We find two differences between the experiments. First, in Experiment 3 low Raven participants imitate the high Raven demonstrator significantly more than the low Raven one when her Raven score is not visible (NovisHigh and NovisLow treatments). This can be explained by the higher amount of effort that low Raven participants put into learning from the actions of the demonstrator when they do not know that their Raven score is low (see Appendix B.6 for the detailed comparison of all analyses). It should be mentioned, though, that the imitation rate of low Raven participants in the NovisHigh treatment is not significantly different in the two experiments. Moreover, Table 6 in Appendix B.6 shows that low Raven participants in Experiment 3, as well as in Experiment 2, do not react to the number of switches of the other. This confirms that high and low Raven participants use different modes of weighing up social and asocial information in both experiments. The second difference we find is that in Experiment 3 participants’ imitation choices are noisier. This can be related to variation in beliefs that participants have about their own ability to perform in the task. Such variability can lead to increased noise in the decisions to imitate as it is less clear to participants how their own competence relates to that of the demonstrator. In fact, this result supports our claim that the information about participants’ own Raven score as well as demonstrator’s is associated with their confidence. The uncertainty about one’s own Raven score in Experiment 3 does not change the results, and the support for our hypotheses is not undermined.

## Discussion

Many studies attempted to identify factors that can explain the diversity of social learning strategies both within and between groups. It was found that many demographic characteristics like, for example, income, ethnicity, and gender, can not explain this variability^32,51–54^. We show that intelligence plays a role in the way people choose to weigh up social and individual information in their decisions. In particular, high fluid intelligence individuals are able to recognize whether the demonstrator is getting high or low payoffs from just observing her actions and without knowing her intelligence. As a result, high fluid intelligence individuals imitate the demonstrator when they deem it worthwhile. Conversely, low fluid intelligence individuals are unable to extract this information from observations and resort to unconditional imitation when they know that the demonstrator has high fluid intelligence. This is in line with the hypothesis that people turn to social learning when they are uncertain about their own ability or, in other words, their level of confidence is low^55,56^. These two modes of processing social information are also related to the findings in studies that explore the degree of reliance on social learning when individual learning is costly or difficult^1,57–60^.

One possible reason why we do find different social learning strategies used by low and high fluid intelligence participants is that in our experiment we did not give explicit information about how hard the task is. When the difficulty of the task is provided by the experimenter^13^, it is plausible to expect that all participants react to it in a similar way. In our case, participants have to learn themselves how difficult the task is for them. Therefore, the choice of how much to rely on social and asocial information depends on participants’ confidence about how to choose in the task and their ability to recognise the competence of the demonstrator. Our findings suggest that these two features are determined by fluid intelligence.

Our results can be interpreted in the light of theories that integrate social and individual learning^1,61–64^. In particular, we find that, at the beginning of the learning task, high fluid intelligence individuals rely on the information about the intelligence of the demonstrator by increasing their imitation (see Fig. 2C). In the rest of the task their imitation rate stops being dependent on this knowledge and is modulated only by the characteristics of the observed behaviour. This is in line with theories that suggest that in the absence of experience, behaviour is driven by social learning, and later the reliance on social learning decreases as individual information accumulates^1,62^. This does not apply to low fluid intelligence individuals who rely on social learning throughout the task when they know that the demonstrator has high intelligence. Thus, we cannot exclude the possibility that low fluid intelligence individuals are unable to integrate the two types of learning^65,66^.

## Methods

The study consisted of three experiments: Experiment 1 in which participants made choices in a 2-armed bandit problem without observing others’ actions, and Experiments 2 and 3 in which participants made choices in the same environment with the only difference that in half the trials they observed the choices made by one of the two participants selected from the first experiment. The purpose of Experiment 1 was to select two participants, one with a high and one with a low Raven score, in order to use them as demonstrators in Experiments 2 and 3. Moreover, we used the data from Experiment 1 to evaluate the improvement in efficiency of the participants in Experiments 2 and 3.

The two demonstrators were chosen using the following procedure. First, we divided participants into deciles of Raven score. Then we calculated the median number of switches between actions for participants in the first and tenth decile. We chose two participants (one in the first and one in the tenth decile) who were the closest to the median. The aim of this procedure was to select two participants who would have a prototypical behaviour in terms of the number of switches in the two extremes of the Raven dimension (the number of switches of the low and high Raven participants are equal to 49 and 20, respectively). We decided to use the number of switches parameter for two reasons: (1) it is an index related to the earnings of the participants and (2) using simulations of optimal behaviour in stationary 2-armed bandit problem we found that the number of switches is an important parameter that can be interpreted as a signal of confidence^44^: sophisticated learners interpret a relatively high number of switches as a signal of bad payoffs and learn to decrease imitation when the number of switches increases.

Participants in Experiments 2 and 3 were divided into four treatments with 2 × 2 design. The dimensions were: (1) the Raven score of the observed other (High or Low) and (2) the information participants received about the Raven score of the observed other (Visible or Non-visible). The Raven score of the observed other could be high (28 correct matrices) or low (15 correct matrices). The treatments are, thus, called VisHigh, VisLow, NovisHigh, and NovisLow. Only participants in the VisHigh and VisLow treatments knew the Raven score of the demonstrator. Participants in the NovisHigh and NovisLow treatments were matched with the corresponding demonstrator without knowing his/her score on the Raven test.

Experiment 2 and Experiment 3 differed in only one respect: in Experiment 2 participants were informed about their own Raven score before the learning task and in Experiment 3 they were not (though, they did take the Raven test before the learning task). Apart from this difference, the experiments were identical.

For the main experiments (Experiments 2 and 3), nine NovisHigh and NovisLow, and ten VisHigh and VisLow sessions were conducted. In each session half the participants observed the high Raven other and the other half observed the low Raven other. All participants were recruited from the subject pool of the Cognitive and Experimental Economics Laboratory at the University of Trento (CEEL). The dates of the sessions and the number of participants per session are reported in Table 9, Appendix G. Summary statistics are provided in Appendix B.1. Non-parametric tests ensure that participants in all experiments come from the same population: no significant differences were found.

On average participants earned about 20.06, in addition to the 3 show-up fee. The presentation of the 2-armed bandit task was performed using a custom made program implemented in Matlab Psychophysical toolbox. The tests and questionnaires were administered with z-Tree software package^67^. A detailed timeline of the experiment and all instructions are reported in Appendix C.

### Experiment 1

51 participants took part in Experiment 1. In the first part of the experiment participants made choices in a 2-armed bandit problem. In the second part they completed a 20-minutes version of the Raven Advanced Progressive Matrices test^68^, the Holt & Laury Risk Aversion test, the Cognitive Reflection Test, and the Empathy Quotient questionnaire^69^, which was added to the study in order to assess whether empathic abilities affect the way participants imitate others. The time-constrained version of the Raven APM test has been shown to be an adequate predictor of the unconstrained Raven APM score^68^.

After entering the lab, participants were randomly assigned to a PC terminal and were given a copy of the instructions (see Appendix C). Instructions were read aloud by the experimenter, and then a set of control questions were provided to ensure the understanding of the 2-armed bandit problem.

The probabilities of getting a 10 cents reward from each of the two hands followed independent stochastic processes (see Fig. 1B). The process is a decaying Gaussian random walk with parameters *λ* = 0.8, decay centre *θ* = 0.5, and Gaussian noise with standard deviation 0.2^70^.

Participants were not aware of how the probabilities change but it was made clear that they would change slowly and independently of their choices, earnings, and each other. The 2-armed bandit task included 200 trials divided into four blocks of approximately 50 trials each. At the end of the task participants were not informed about their earnings until after they completed the second part of the experiment (participants were not told their *total* earnings at the end of the choice task, though, in principle, they could have calculated it by observing the outcomes after each trial). In the second part of the experiment, participants where provided with 20 minutes version of the Raven Advanced Progressive Matrices test. They were told that they have 20 minutes to solve as many problems as they can and that they would earn 30 cents for each correct answer. If participants did not complete an item or their answer was incorrect they would earn 0 cents for that item. At the end of the Raven test participants completed the Holt and Laury lottery task (with real incentives, see Appendix E), the CRT test and the EQ questionnaire (Appendices C and F). There was no time limit to complete these three tasks and no payment was provided for the CRT test, and the EQ questionnaire. At the end of the Holt & Laury task a single lottery was selected at random and played by the computer to determine payment.

At the end of the second phase, participants were paid according to their choices in the 2-armed bandit problem, their performance in the Raven problems, the outcome of the selected lottery, and a show-up fee of € 3.

### Experiments 2 and 3

In Experiments 2, 160 participants first completed the 20-minute version of Raven APM test, the Holt & Laury Risk Aversion test, the Cognitive Reflection Test, and the Empathy Quotient questionnaire, and then played in the 2-armed bandit task. Before the learning task they were informed about their own Raven score. The only difference with Experiment 1 was that participants in the 2-armed bandit problem, sometimes, and before making their choices, also observed the choices (but not the outcomes) made by one of the two selected participants from Experiment 1. The choices of the demonstrator were provided in half the trials (in 100 out of the 200 trials) between trial 10 and trial 200 in blocks of randomized length of 6 to 12 consecutive trials. It was made clear to participants that the observed behaviour was from a real person who took part in the experiment approximately one month before and that he/she chose in the same exact environment (the probabilities of reward in each period were identical). Participants knew that the observed other has completed all parts of the experiment, including the Raven test that they completed at the beginning of the experiment. They were also informed that the demonstrator did not herself observe anyone while choosing in the 2-armed bandit problem task.

Experiment 3 had 142 participants and was the same as Experiment 2, only participants were not informed about their Raven score before the learning task. Also, participants in Experiment 3 did not complete the Holt & Laury Risk Aversion test, the Cognitive Reflection Test, and the Empathy Quotient questionnaire.

In Experiments 2 and 3 participants were shown (and explained) a histogram of the number of the Raven APM problems solved by the 51 participants from Experiment 1 (see Fig. 9 version A in Appendix C). In this way, in Experiment 2 they had the possibility to compare their score in the Raven test, which they knew before starting the 2-armed bandit task, with that of the group from which the demonstrator was chosen. No information about a possible connection between performance in the Raven test and the learning task was provided.

The instructions were identical for all participants, except for the information that was given about the score obtained by the observed other. In the NovisHigh and the NovisLow treatments the score obtained by the observed other remained unknown (only the distribution of all Raven score was known). Conversely, in the VisHigh and the VisLow treatments the score of the observed other was marked in red on the histogram and shown on the screen during the experiment (see Fig. 9 versions B and C in Appendix C).

### Data availability

The data are available upon request.

### Ethics committee

The study was approved by the Human Research Ethics Committee of the University of Trento (http://www.unitn.it/en/ateneo/1755/human-research-ethics-committee).

### Informed consent

All participants gave informed consent to take part in the experiment.

### Guidelines and Regulations

All experiments were carried out in accordance with relevant guidelines and regulations of the Human Research Ethics Committee of the University of Trento (http://www.unitn.it/en/ateneo/1755/human-research-ethics-committee).

### Experimental protocol

The experimental protocol was approved by the Human Research Ethics Committee of the University of Trento (http://www.unitn.it/en/ateneo/1755/human-research-ethics-committee).

### Images Used in the Experimental Design

All images used in the experimental design (Fig. 1 and all figures in Appendix C) were drawn by the authors and are not subject to any copyright. In particular, the drawing of a person was drawn by the authors and the picture of a coin was obtained by scanning a real 10 cents coin.

## Electronic supplementary material

Supplementary Material
