# Supplementary material for: The Role of Intelligence in Social Learning
Source: Sci Rep. 2018 May 2;8:6896. doi: 10.1038/s41598-018-25289-9 (PMC5932062; doi:10.1038/s41598-018-25289-9)
Supplement: Supplementary file 1 — Supplementary Material [file 41598_2018_25289_MOESM1_ESM.pdf]

The Role of Intelligence in Social Learning

Alexander Vostroknutov Luca Polonio Giorgio Coricelli

Supplementary Material (for online publication)

A Variables

Variable	Range	Definition
imitation	0/1	0 if participant chose different action than the other in a given period, 1 if he chose the same action
adjusted imitation	$[0, 1]$	for each participant, the average rate of imitation in periods when the other is observed minus the average rate of imitation when the other is not observed
value of imitation	$[0, 1]$	for each period t, the average payoff from choosing the same action as the observed other for the last 10 times in the past
switches of other	$[0, 1]$	for each period t, the average number of switches of the observed other in the last 10 periods (when observed only)
is efficient	0/1	for each period t, is 1 if the choice was efficient (the action with highest actual probability of reward was chosen)
visible	0/1	for each participant, is 1 if participant is in VisHigh or VisLow treatments
obshigh	0/1	for each participant, is 1 if participant is observing high Raven other
ravterc2	0/1	for each participant, is 1 if she is in the second tercile of Raven scores
ravterc3	0/1	for each participant, is 1 if she is in the third tercile of Raven scores
number of switches	$[0, 1]$	number of switches from one action to another that a participant made throughout the experiment. Normalized to $[0, 1]$ from $[0, 200]$
distance from optimum	$[0, 1]$	the number of periods in which a participant did not choose efficient action. Normalized to $[0, 1]$ from $[0, 200]$
raven score	$[0, 1]$	the value of Raven score of a participant normalized to $[0, 1]$
earnings	$[0, 1]$	the amount earned by participants in the bandit game. Normalized to $[0, 1]$ from $[9.1, 14.7]$

Table 2: Variables used in the analyses and regressions.

B Additional Analyses

B.1 Summary Statistics

We report the summary statistics for Experiments 1-3. Table 3 shows the scores obtained in the tests we conducted (average and standard error) and the demographic information.

	Other Observed	Visibility of Raven Other	Visibility of Raven Own	Other's Raven	Own Raven	CRT	HL	EQ	Male	Age
**Experiment 1**	No		No		21.51 (0.52)	1.46 (0.19)	5.82 (0.33)	40.65 (1.67)	0.57 (0.08)	22.81 (0.43)
**Experiment 2**					20.93 (0.35)	1.32 (0.09)	6.02 (0.13)	41.83 (0.66)	0.52 (0.04)	23.17 (0.23)
NovisLow	Yes	No	Yes	Low	20.53 (0.70)	1.46 (0.18)	6.20 (0.27)	42.40 (1.31)	0.54 (0.08)	23.27 (0.43)
VisLow	Yes	Yes	Yes	Low	21.48 (0.60)	1.38 (0.19)	5.82 (0.26)	42.21 (1.25)	0.57 (0.08)	23.05 (0.40)
NovisHigh	Yes	No	Yes	High	21.50 (0.72)	1.24 (0.19)	5.81 (0.28)	41.49 (1.22)	0.49 (0.08)	22.76 (0.43)
VisHigh	Yes	Yes	Yes	High	20.27 (0.77)	1.20 (0.17)	6.23 (0.19)	41.27 (1.43)	0.50 (0.08)	23.54 (0.47)
**Experiment 3**					22.10 (0.34)				0.50 (0.04)	22.11 (0.17)
NovisLow	Yes	No	No	Low	21.47 (0.64)				0.50 (0.09)	22.00 (0.36)
VisLow	Yes	Yes	No	Low	22.19 (0.78)				0.62 (0.08)	22.13 (0.36)
NovisHigh	Yes	No	No	High	22.28 (0.64)				0.46 (0.08)	22.21 (0.36)
VisHigh	Yes	Yes	No	High	22.44 (0.67)				0.42 (0.08)	22.08 (0.29)

Table 3: Summary statistics for all data. Standard errors in parentheses.

The Kolmogorov-Smirnov tests were used to test the hypotheses that the distributions of the Raven, CRT, HL, and EQ scores are different between the experiments. None of the comparisons was found to be statistically significant, thus, supporting the hypothesis that participants in the experiments come from the same population. This is important since participants in Experiments 2 and 3 were presented the distribution of the Raven scores of participants from Experiment 1 and absence of significant differences means that they could compare their score with that of the observed other in a reliable way.

B.2 Regression Analysis of the Adjusted Imitation Rate

To analyse the adjusted rate of imitation we run the regressions reported in Table 4. The dependent variable is the adjusted rate of imitation (adjusted imitation). The independent variables are the indicators for visibility of the demonstrator's Raven score (visible), the demonstrator being high Raven (obshigh), the terciles of Raven score of the participants (ravter2 and ravterc3), and all the interactions. The following results are mentioned in the main text for Experiment 2 (Column 1 of Table 4). The differences in coefficients between the high and the low Raven other are significant for all terciles and both a visible and non-visible demonstrator's Raven score except for low Raven participants (tercile 1) when the demonstrator's Raven score is not visible. These differences are represented by the following linear combinations of the coefficients (from left to right on Figure 2A): obshigh + obshigh·ravterc2 (0.108**, $p = 0.003$); obshigh + obshigh·ravterc3 (0.162**, $p = 0.004$); obshigh + visible·obshigh (0.254***, $p < 0.001$); obshigh + visible·obshigh + obshigh·ravterc2 + visible·obshigh·ravterc2 (0.214***, $p < 0.001$); obshigh + visible·obshigh + obshigh·ravterc3 + visible·obshigh·ravterc3 (0.084*, $p = 0.033$).

	adjusted imitation	
	Experiment 2	Experiment 3
visible	–0.082*	0.008
	(0.038)	(0.044)
obshigh	0.075	0.120**
	(0.044)	(0.045)
visible·obshigh	0.179**	0.058
	(0.059)	(0.063)
ravterc2	–0.012	–0.027
	(0.033)	(0.042)
ravterc3	–0.031	0.009
	(0.052)	(0.045)
visible·ravterc2	0.073	0.023
	(0.054)	(0.058)
visible·ravterc3	0.128	–0.056
	(0.067)	(0.060)
obshigh·ravterc2	0.034	0.040
	(0.057)	(0.062)
obshigh·ravterc3	0.087	0.042
	(0.071)	(0.058)
visible·obshigh·ravterc2	–0.074	–0.035
	(0.083)	(0.085)
visible·obshigh·ravterc3	–0.257**	0.018
	(0.090)	(0.081)
constant	0.025	0.017
	(0.023)	(0.034)
N participants	160	142

Table 4: OLS regressions of the adjusted imitation rate. Errors are robust. Standard errors in parentheses. * – $p < 0.05$; ** – $p < 0.01$.

Participants with low and middle Raven scores (terciles 1 and 2) imitate significantly more when they know the Raven score of the high Raven demonstrator than when they do not know it. The differences in the coefficients are given by visible + visible·obshigh (0.097*, $p = 0.032$) for low Raven participants and by visible + visible·obshigh + visible·ravterc2 + visible·obshigh·ravterc2 (0.097*, $p = 0.034$) for middle Raven participants. The same comparison for high Raven participants is insignificant.

B.3 The Cognitive Reflection Test

The Cognitive Reflection Test (CRT, Frederick, 2005) was designed for somewhat different purpose than the Raven test. It measures the tendency to implement one of the two types of cognitive processes: those that are slower and more reflective and those executed rapidly with little conscious deliberation. In particular, a high CRT score reflects an ability to inhibit an intuitive and wrong response in favor of deeper reasoning about the characteristics of the problem. There might be two reasons why we observe the equivalent results with CRT and Raven tests: 1) in our data the CRT and Raven scores are correlated (Spearman's $\rho = 0.3558$, $p < 0.0001$); 2) both high Raven and CRT participants overcome the desire to simply imitate the high Raven other and instead search for patterns in her choices.

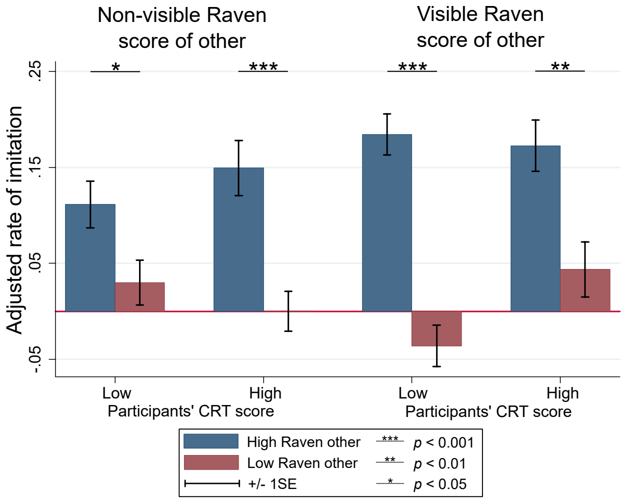

Figure 4: The adjusted rate of imitation in the four treatments by the median of the CRT score. The significance of the differences in coefficients of the linear regression between observing the high and low Raven others are shown on the graph (the leftmost $p < 0.02$, all other $p < 0.002$). The imitation rates of low Raven participants between the conditions when the Raven score of the high Raven other is observed and not is significant (coefficient difference $p = 0.027$).

If we divide participants according to the CRT instead of Raven, we observe very similar patterns. Figure 4 shows adjusted imitation of participants in the four treatments averaged over the two groups: below median and above median score on the CRT test. The conclusions are the same, but slightly weaker. Participants in low and high CRT groups imitate the high Raven other more regardless of the visibility of her Raven score. The differences in the coefficients between the low and high Raven other for the four groups (from left to right) are: 0.081^{*}, $p = 0.017$; 0.149^{***}, $p < 0.001$; 0.220^{***}, $p < 0.001$; 0.129^{**}, $p = 0.001$. For low CRT participants the difference in the imitation rates of high Raven other when her Raven score is known and when it is not is significant (0.073^{*}, $p = 0.027$).

B.4 Additional Analyses for Section "Efficiency and Earnings"

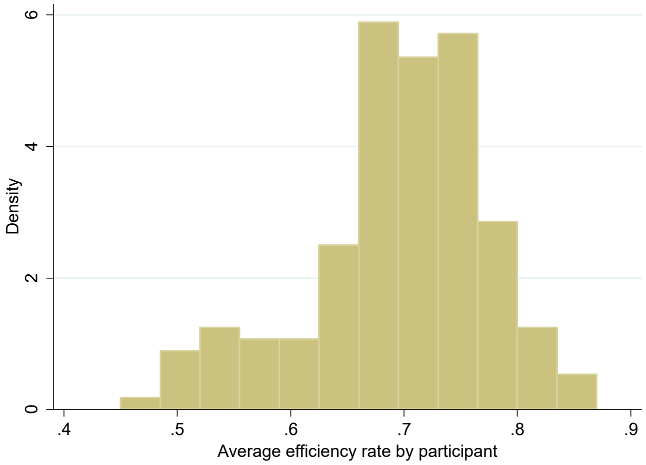

Figure 5: The distribution of the average efficiency rates for each participant (Experiment 2). The average is taken over the indicator variable is efficient which is 1 if the action with the highest probability of reward was chosen.

B.5 Efficiency and Behaviour

In this subsection we explore the mechanism responsible for the difference in efficiency improvements of low and high Raven participants in Experiment 2. The regression in Column 1 of Table 5 shows that the Raven score and the total number of switches that participants make are negatively correlated. In addition, the number of switches determines how far the choices of participants are from the most efficient strategy (Column 2): the more participants switch the less efficient their strategy is. The same holds for the earnings. More switches predicts less money earned (Column 3). We also see that the Raven score predicts the amount of money earned (Column 4). These correlations suggest that Raven score, the number of switches, and efficiency/earnings are intimately related. In particular, high Raven participants switch less and also increase their imitation when the demonstrator switches less, which together leads to the increase in the efficiency and earnings.

	(1)	(2)	(3)	(4)
Dependent variable:	number of switches	distance from optimum	earnings	earnings
raven score	–0.162* (0.063)			0.203* (0.100)
number of switches		0.532*** (0.058)	–1.013*** (0.124)	
constant	0.358*** (0.046)	0.178*** (0.016)	0.782*** (0.034)	0.394*** (0.074)
N participants	158	160	160	158

Table 5: OLS regressions of switchiness, distance from optimal policy and amount earned by treatment in Experiment 2. Errors are robust. Standard errors in parentheses. * – $p < 0.05$; ** – $p < 0.01$; *** – $p < 0.001$.

To support these findings further, we analyse what information gets transferred from the demonstrators to participants and whether they learn correctly from observations. We look at the choices of the participants in Experiment 1. The number of switches is correlated with the Raven score (Spearman's $\rho = -0.335$, $p = 0.016$), and the earnings are correlated with the number of switches (Spearman's $\rho = -0.329$, $p = 0.018$). We have established that high Raven participants increase imitation when they observe the other reducing the number of switches. Given that lower number of switches is correlated with higher Raven score, we can conclude that high Raven participants imitate the other the more, the higher Raven score the other has. In this sense, the information about the Raven score of the observed other gets transferred by observing the number of switches. This is also efficient, because a lower number of switches is associated with higher earnings.

B.6 Comparison of Experiments 2 and 3

In this Appendix we compare the behaviour of our participants in Experiments 2 and 3, which differ in the availability of information about one's own Raven score. We start with the main graphical results as in Figure 2 in the main text. Figure 6 shows the analysis of the adjusted imitation rate in Experiment 3.

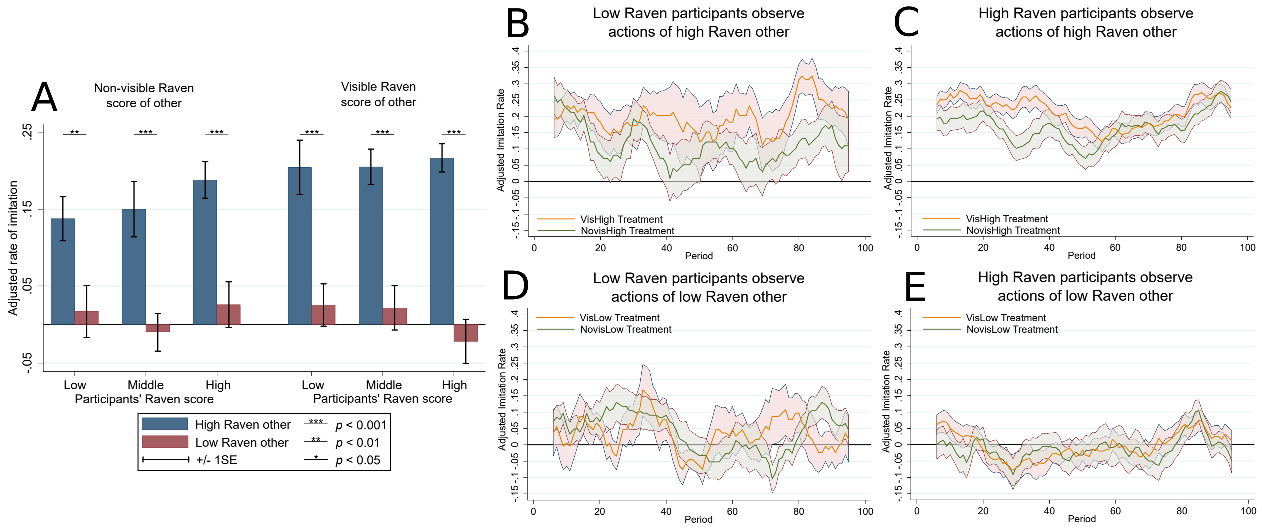

Figure 6: The adjusted imitation rate in Experiment 3. p-values represent the differences in coefficients coming from the regression in Table 4 (right column).

Panels A of both figures look very similar. In particular, participants in both experiments imitate the high Raven other significantly more than the low Raven other, regardless of the visibility of the other's Raven score. The only difference between Experiments 2 and 3 is that low Raven participants (tercile 1) in Experiment 3 show a significant increase in imitation of the high Raven demonstrator, as compared to the low Raven one, when her score is not visible (the NovisLow vs. NovisHigh treatments). This result is in line with the theoretical and experimental literature on contests (see Sheremeta, 2013, for review) which shows that players who have a belief that their cost of learning is too high choose not to put effort into winning the contest. Similarly, in our experiment when we inform participants that they have a low Raven score they might choose to put less effort into learning from the actions of the demonstrator since it makes them believe that they will not be able to learn anyway. It is also true that in Experiment 3 the adjusted imitation of the high Raven other looks slightly higher overall across all the terciles of participants' own Raven scores than in Experiment 2. However, these differences are not significant if tested by terciles, treatments, and overall.[1] When comparing the imitation rates between treatments with a visible and not visible Raven score of the demonstrator, we find that low and middle Raven participants in Experiment 2 do increase their imitation when they know that they observe the high Raven other as compared to the situation when they do not (difference in coefficients 0.097*, $p = 0.032$ and 0.097*, $p = 0.034$). In Experiment 3, low and middle Raven participants do not significantly increase their imitation when the other's Raven score is visible (all $p > 0.146$), which might be due to their increasing the effort to learn from the actions of the demonstrator as we proposed above. In addition, low and middle Raven participants might not increase their imitation in the VisHigh treatment as compared to the NovisHigh treatment because they think that their Raven score is similar to the score of the high Raven demonstrator (overconfidence about own ability, Svenson, 1981; Malmendier and Tate, 2005). This does not happen in Experiment 2 since participants know that their Raven score is lower than that of the high Raven demonstrator.

When looking at the graphs with moving averages of the adjusted imitation (Panels BCDE, Figures 2 and 6), the similarities are also evident. In particular, in both experiments, observing the high Raven

[1]Except for one test of the third tercile in the VisHigh treatment, the right-most blue bar (rank-sum $p = 0.0101$). Though, this comparison becomes insignificant when Bonferroni corrected for 9 tests performed.

	Prob[imitation = 1]			
	Experiment 2		Experiment 3	
Participants:	Low Raven	High Raven	Low Raven	High Raven
visible	0.057	0.274	0.415	–0.419*
	(0.317)	(0.238)	(0.301)	(0.213)
value of imitation	1.011***	1.209***	1.405***	0.789***
	(0.236)	(0.215)	(0.234)	(0.181)
switches of other	–0.546	–0.664*	–0.254	–0.504***
	(0.351)	(0.304)	(0.165)	(0.118)
visible × value of imitation	0.118	–0.356	–0.625	0.829***
	(0.346)	(0.281)	(0.361)	(0.251)
visible × switches of other	0.216	0.002	0.006	–0.051
	(0.510)	(0.413)	(0.250)	(0.163)
constant	0.388	0.281	0.167	0.699***
	(0.222)	(0.178)	(0.197)	(0.154)
N observations	6,095	8,463	4,016	9,853
N participants	67	93	41	101

Table 6: Random effects logit regression of imitation choices separately for Experiment 2 and 3 data. Standard errors in parentheses. * – $p < 0.05$; ** – $p < 0.01$; *** – $p < 0.001$.

other (Panels BC) makes imitation higher than when observing the low Raven other (Panels DE), which is a reiteration of the results just described. The dynamics in Panels DE looks pretty much the same in the two experiments. In Panels C it should be noted that high Raven participants imitate the high Raven other significantly more in the VisHigh treatment for longer number of periods in Experiment 3 (for around 55 periods) than in Experiment 2 (for around 25 periods). In Panels B we see that imitation in the VisHigh treatment is always higher than in the NovisHigh treatment in both experiments. However, this difference is only significant in Experiment 2 (for around 50 periods). Both differences in the results can be accounted for by the difference in the design between Experiments 2 and 3: in Experiment 3 participants do not know their own Raven score, which makes them less *confident* about whether to follow the other or try to think for themselves. High Raven participants follow the high Raven other significantly more and for longer time since they are unsure if they would perform better or worse without observation. Similarly, for low Raven participants, the variance of imitation choices is much higher in Experiment 3, which points towards uncertainty in judgement about how to treat the observations of the other. Overall, though, the differences between the two experiments are minor and general patterns of behaviour do not change, which allows us to conclude that the information about one's own Raven score does not conceptually change the way participants act in the experiment.

Next, we compare the regressions that predict the imitation rate. Table 6 presents the regressions for the two experiments. The exact same conclusions as for the aggregate data can be made for each experiment. The variable value of imitation is significant in all regressions for both low and high Raven participants. The variable switches of other is only significant for high Raven participants. The interactions with the visibility of the Raven score of the other are not changing the significance levels (when adding coefficients from the baseline and interaction terms). All this also holds for the linear models in Table 7. Therefore, this analysis shows the same behaviour in both experiments.

Table 8 shows the same regressions as in Table 5 only for Experiment 3. All the coefficients and significance levels are almost the same. This suggests the same connection between the Raven score, the number of switches, and earnings as in Experiment 2.

	imitation			
	Experiment 2		Experiment 3	
Participants:	Low Raven	High Raven	Low Raven	High Raven
visible	0.010	0.057	0.085	–0.080*
	(0.058)	(0.047)	(0.061)	(0.040)
value of imitation	0.202***	0.238***	0.289***	0.161***
	(0.045)	(0.042)	(0.047)	(0.036)
switches of other	–0.103	–0.130*	–0.051	–0.098***
	(0.067)	(0.060)	(0.033)	(0.023)
visible×value of imitation	–0.004	–0.070	–0.132	0.149**
	(0.065)	(0.055)	(0.072)	(0.048)
visible×switches of other	0.055	–0.001	0.004	–0.004
	(0.096)	(0.081)	(0.049)	(0.031)
constant	0.592***	0.576***	0.550***	0.656***
	(0.041)	(0.035)	(0.040)	(0.029)
N	6,095	8,463	4,016	9,853
N participants	67	93	41	101

Table 7: Random effects OLS regressions of imitation choices. Standard errors in parentheses. * – $p < 0.05$; ** – $p < 0.01$; *** – $p < 0.001$.

	(1)	(2)	(3)	(4)
Dependent variable:	number of switches	distance from optimum	earnings	earnings
raven score	–0.177**			0.268**
	(0.060)			(0.096)
number of switches		0.481***	–0.790***	
		(0.050)	(0.101)	
constant	0.383***	0.187***	0.705***	0.308***
	(0.046)	(0.014)	(0.028)	(0.073)
N participants	142	142	142	142

Table 8: OLS regressions of switchiness, distance from optimal policy and amount earned by treatment in Experiment 3. Errors are robust. Standard errors in parentheses. * – $p < 0.05$; ** – $p < 0.01$; *** – $p < 0.001$.

C Experimental Instructions

The following is a translation of the original instructions in Italian. The experimenter read the instructions aloud to participants while they followed along their own copy. Original instructions are available upon request. We decided to leave the instructions as is, which means that the numbering of figures in the instructions goes separately from the main text. In what follows, the numbers of figures refer *only* to figures in the instructions.

We report only the instructions for the main task since for other tasks (Raven test and Holt and Laury task) we use standard procedures. We do not report the instructions for Experiment 1 since they are the same as those below only without the observational part and presentation of Raven histograms.

INSTRUCTIONS

Dear student you are about to participate in an experiment on decision making. Your privacy is guaranteed: results will be used and published anonymously. Your earnings will depend on your performance in the experiment, according to the rules which we will explain to you shortly. You will be paid privately at the end of the experimental session. Other participants will not be informed about your earnings. The maximum amount you can earn in the experiment is €35.85 and the minimum is €3.10.

The experiment is divided in 3 parts. Each part of the experiment will be described in detail below.

Part One
COMPLETION OF MATRICES PROBLEMS
See Appendix D.

Part Two
QUESTIONNAIRES
See Appendix E, F, and G.

Part Three

CHOICE TASK

In this part of the experiment you will face 200 trials divided in four blocks of 50 trials each. On each trial you will be asked to choose between two symbols. The two symbols are like two slot machines that give you a reward of 10 cents with a certain—unknown—probability (0 otherwise). You do not have to pay before to choose your symbol and your goal will be to try to choose the symbol that gives you the 10 cents with the highest probability. However, you do not know the probability associated with the two symbols of getting the 10 cents, and you will need to figure out what is the most convenient symbol to play following the feedback you will receive from time to time. In fact, after choosing one of the two symbols you will be notified about the outcome of your bet (if you have won 10 cents or 0 cents).

The experiment proceeds as follows:
When a red silhouette appears on the screen (Figure 1), it means that you have to choose one of the two symbols. After you press the answer button your choice will appear under the selected symbol (Figure 2) and you will receive a feedback about the outcome of your choice (Figure 3 appears if you have won 0 cents and figure 4 if you have won 10 cents).

Figure 1

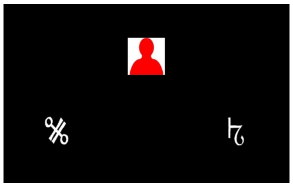

Figure 2

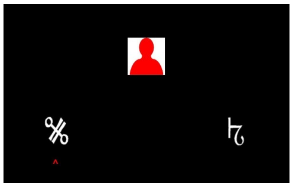

Figure 3

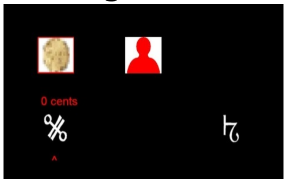

Figure 4

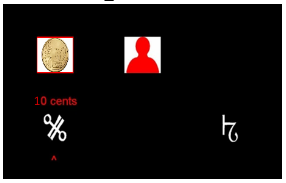

Probability of winning associated with the two symbols
At the beginning of the experiment a certain probability of winning will be associated with each of the two symbols. The probability of winning will change slowly in the course of the experiment (Figure 5). For example, suppose that at the beginning of the experiment the probability of winning 10 cents associated with the symbol X is equal to 0.8 (you receive the reward 8 times out of 10), this probability may increase or decrease slightly in the subsequent trials until the end of the experiment. **However, keep in mind that Figure 5 has only a descriptive purpose and it does not show the exact time course of the probabilities. Moreover, remember that the probabilities of winning associated with the two symbols are independent; therefore, if the probability of winning associated with one symbol increases from one trial to the next, the probability of winning associated with the other symbol does not necessarily decrease.**

Figure 5

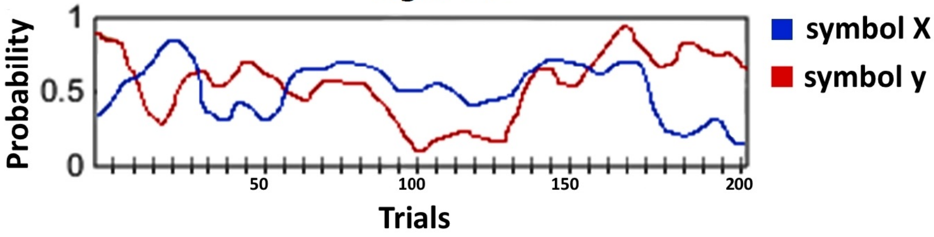

Observational phase
In addition to the feedback about the outcome of your choice, you will receive additional information during the experiment. Before making your choice, you often have the chance to observe the choice made (in the same trial) by a participant carrying out this same experimental task. The only difference between you and this participant is that he/she could not observe the actions of another participant. This participant will be represented with a green silhouette (Figure 6) and from time to time, before making your choice, you will see the choice made by this participant in the trial you are about to play (Figure 7). After you have observed the choice of this participant, you will make your decision.

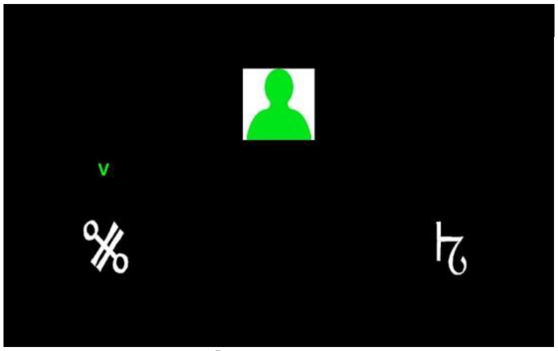

Figure 6

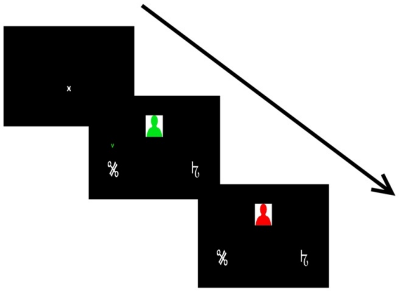

Figure 7

Timeline of the experimental phases
At the beginning of the experiment you will not see the choices made by the other participant. Then, in different time phases, and always before making your choice, you will see the choice made by the other participant. The other participant had the same goal as you, which is to choose the option that would guarantee the highest probability of winning.

In Figure 8 you can see a diagram that summarizes the experimental structure. Importantly, this is

just an example for illustration only, Figure 8 does not show the real probabilities of winning associated to the two options and the exact time course of the experiment.

Figure 8

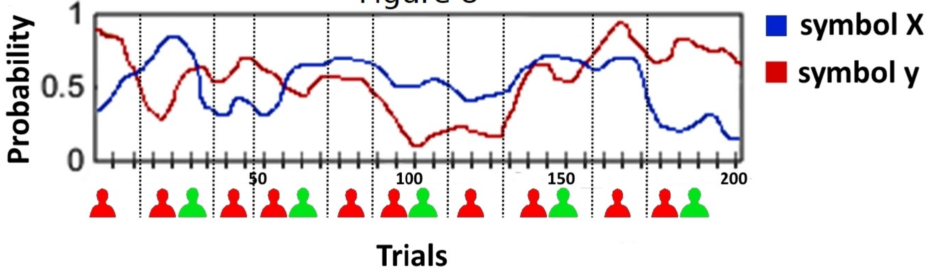

Random selection of the observed other (Non-visible condition)

The participant you will observe has completed (like you) all the parts of the experiment, including the questionnaire on matrices problems that you completed at the beginning of the experimental session. The chart below (Figure 9) shows the scores obtained by all participants who participated in this study in the previous sessions (December 2015). The participant that you will observe was selected among these 51 participants.

Figure 9 (version a)

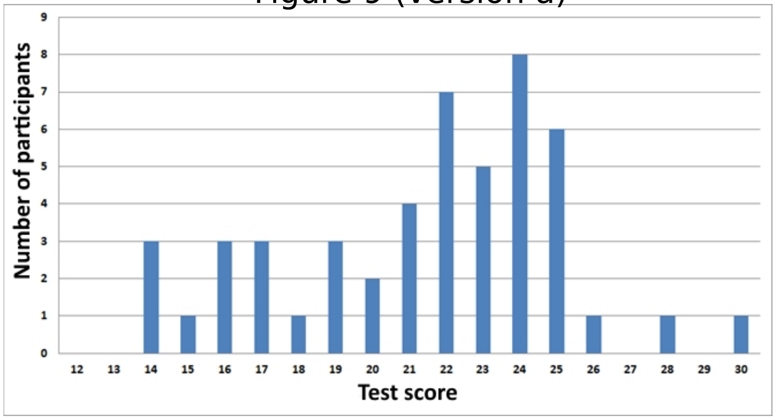

Random selection of the observed other (Visible low condition)

The participant you will observe has completed (like you) all the parts of the experiment, including the questionnaire on matrices problems that you completed at the beginning of the experimental session. The chart below (Figure 9) shows the performance achieved by all participants who participated in this study in the previous sessions (December 2015). The score achieved by the participant that you will observe is coloured in red.

Figure 9 (version b)

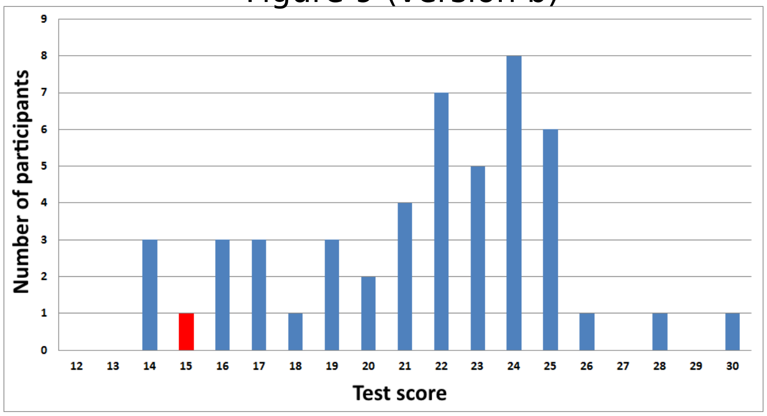

Random selection of the observed other (Visible high condition)

The participant you will observe has completed (like you) all the parts of the experiment, including the questionnaire on matrices problems that you completed at the beginning of the experimental session. The chart below (Figure 9) shows the performance achieved by all participants who participated in this study in the previous sessions (December 2015). The score achieved by the participant that you will observe is coloured in red.

Figure 9 (version c)

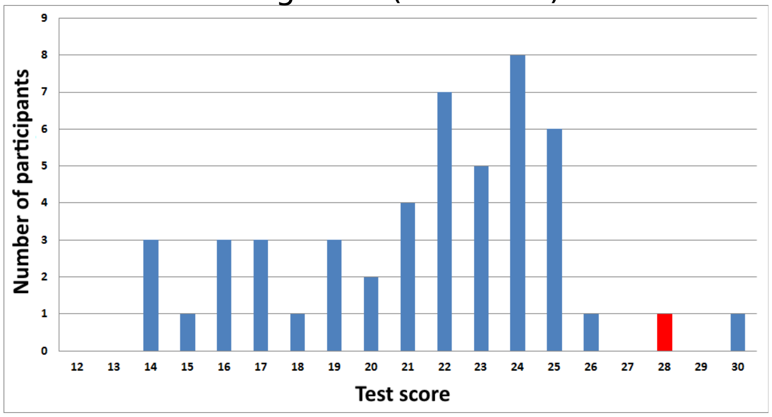

D The Raven Test

In this part participants were presented with the 20-minute Raven test (Raven *et al.*, 1998; Hamel and Schmittmann, 2006). Figure D shows an example problem.

1.

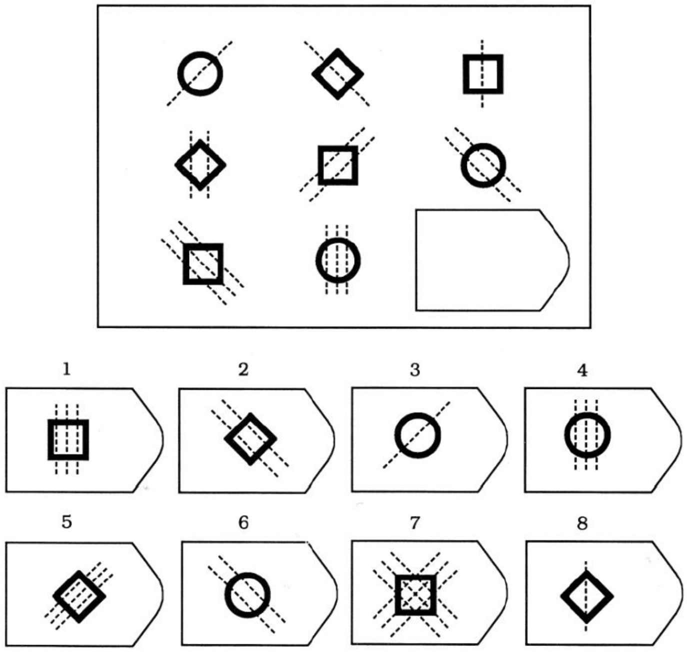

E Holt and Laury Lotteries

The following figure shows how the Holt and Laury task was presented to participants. The payoffs were identical to the payoffs in Holt and Laury (2002). Participants did not know how much they earned until the end of the experiment.

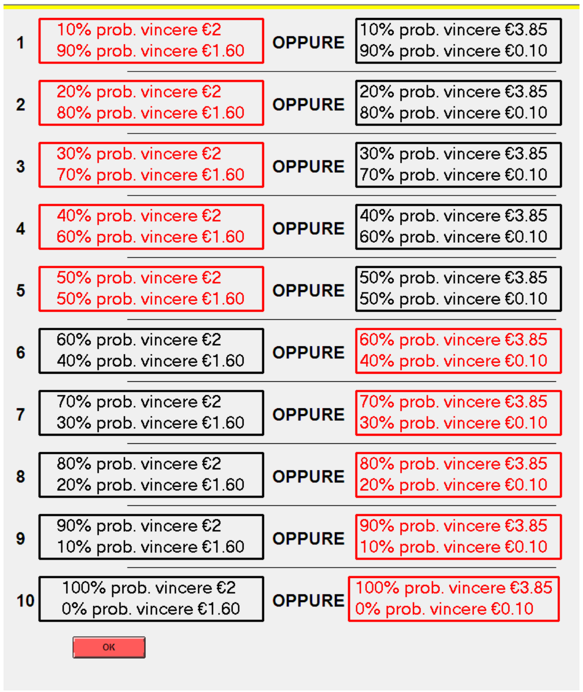

F Empathy Quotient

The Empathy Quotient consists of 60 questions aimed at assessing autistic traits. It is designed for healthy adults (Baron-Cohen and Wheelwright, 2004).

The questions can be found here: http://docs.autismresearchcentre.com/tests/EQ.pdf.

G Details of the Experiment

	**Date**	N **Participants**
**Experiment 1**		
	Dec 11, 2015	21
	Dec 14, 2015	16
	Dec 18, 2015	21
**Experiment 2**		
NovisHigh and NovisLow		
	Jan 22, 2016	22
	Jan 22, 2016	20
	Feb 16, 2016	20
	Feb 16, 2016	12
VisHigh and VisLow		
	Jan 29, 2016	20
	Jan 29, 2016	12
	Feb 04, 2016	18
	Feb 04, 2016	20
	Feb 17, 2016	4
	Feb 17, 2016	14
**Experiment 3**		
NovisHigh and NovisLow		
	Jan 17, 2018	13
	Jan 17, 2018	12
	Jan 18, 2018	4
	Jan 18, 2018	19
	Jan 18, 2018	23
VisHigh and VisLow		
	Jan 16, 2018	15
	Jan 16, 2018	20
	Jan 16, 2018	23
	Jan 17, 2018	15

Table 9: Summary of experimental sessions.

References

Baron-Cohen, S. and Wheelwright, S. (2004). The empathy quotient: An investigation of adults with asperger syndrome or high functioning autism, and normal sex differences. *Journal of Autism and Developmental Disorders*, **34** (2), 162–175.

Frederick, S. (2005). Cognitive reflection and decision making. *Journal of Economic Perspectives*, **19** (4), 25–42.

Hamel, R. and Schmittmann, V. D. (2006). The 20-minute version as a predictor of the raven advanced progressive matrices test. *Educational and Psychological Measurement*, **66** (6), 1039–1046.

Holt, C. A. and Laury, S. K. (2002). Risk aversion and incentive effects. *American Economic Review*, **92** (5), 1644–1655.

Malmendier, U. and Tate, G. (2005). Ceo overconfidence and corporate investment. *The journal of finance*, **60** (6), 2661–2700.

Raven, J., Raven, J. C. and Court, J. H. (1998). The advanced progressive matrices. In *Manual for Ravens Progressive Matrices and Vocabulary Scales*, Oxford, England: Oxford Psychologists Press/San Antonio, TX: The Psychological Corporation.

Sheremeta, R. M. (2013). Overbidding and heterogeneous behavior in contest experiments. *Journal of Economic Surveys*, **27** (3), 491–514.

Svenson, O. (1981). Are we all less risky and more skillful than our fellow drivers? *Acta psychologica*, **47** (2), 143–148.
